# Tree Rings Show Recent High Summer-Autumn Precipitation in Northwest Australia Is Unprecedented within the Last Two Centuries

**DOI:** 10.1371/journal.pone.0128533

**Published:** 2015-06-03

**Authors:** Alison J. O'Donnell, Edward R. Cook, Jonathan G. Palmer, Chris S. M. Turney, Gerald F. M. Page, Pauline F. Grierson

**Affiliations:** 1 Ecosystems Research Group, School of Plant Biology, The University of Western Australia, Crawley, Western Australia, Australia; 2 Lamont-Doherty Earth Observatory, Columbia University, Palisades, New York, United States of America; 3 Climate Change Research Centre, School of Biological, Earth and Environmental Sciences, University of New South Wales, Sydney, New South Wales, Australia; University of California San Diego, UNITED STATES

## Abstract

An understanding of past hydroclimatic variability is critical to resolving the significance of recent recorded trends in Australian precipitation and informing climate models. Our aim was to reconstruct past hydroclimatic variability in semi-arid northwest Australia to provide a longer context within which to examine a recent period of unusually high summer-autumn precipitation. We developed a 210-year ring-width chronology from *Callitris columellaris*, which was highly correlated with summer-autumn (Dec–May) precipitation (r = 0.81; 1910–2011; *p* < 0.0001) and autumn (Mar–May) self-calibrating Palmer drought severity index (scPDSI, r = 0.73; 1910–2011; *p* < 0.0001) across semi-arid northwest Australia. A linear regression model was used to reconstruct precipitation and explained 66% of the variance in observed summer-autumn precipitation. Our reconstruction reveals inter-annual to multi-decadal scale variation in hydroclimate of the region during the last 210 years, typically showing periods of below average precipitation extending from one to three decades and periods of above average precipitation, which were often less than a decade. Our results demonstrate that the last two decades (1995–2012) have been unusually wet (average summer-autumn precipitation of 310 mm) compared to the previous two centuries (average summer-autumn precipitation of 229 mm), coinciding with both an anomalously high frequency and intensity of tropical cyclones in northwest Australia and the dominance of the positive phase of the Southern Annular Mode.

## Introduction

Change in global precipitation patterns and the frequency and duration of droughts is likely to have direct and significant socioeconomic and ecological consequences, yet is arguably one of the least understood consequences of climate change [1]. For Australia, the driest inhabited continent in the world, potential impacts of changing precipitation patterns are a major concern. Australian precipitation is highly variable on inter-annual and decadal timescales [2], particularly so in the semi-arid and arid interior, which is characterised by prolonged drought periods interrupted by episodic intense precipitation events. Model projections suggest that variability in precipitation will become more extreme across Australia, resulting in an increased frequency and duration of dry periods interspersed with more-intense precipitation events [3]. An Increase in the frequency and/or intensity of extreme hydroclimatic events would have the potential to increase the frequency and severity of floods, droughts, and wildfires, with implications for agriculture, forestry, mining, water quality, insurance risk and infrastructure [3].

Significant shifts in precipitation have already been observed across parts of Australia over recent decades. Of particular note is an increase in summer-autumn precipitation in the semi-arid and arid northwest since the 1960s [4], while winter-spring precipitation has declined in the southwest [5]. However, there is considerable uncertainty surrounding the significance of recent observed trends in precipitation and the accuracy of model projections for Australia [6]. Short instrumental records (often <100 years) make it difficult to understand long-term natural variability and how recent trends or shifts fit within the full natural range of variability. Importantly, decadal-scale natural variability in Australian precipitation is comparable in magnitude to projected greenhouse-forced changes (generally -10 to +5% change), which could mask or significantly enhance any trend that may be driven by elevated greenhouse gas concentrations [3]. Developing long-term records of past climate from proxy data sources is therefore critical for understanding the natural range of variability of the Australian climate system and identifying potential trends or shifts outside of the natural range.

Reconstructions of past hydroclimate from proxy data sources (e.g., corals, stalagmites, sediments, and tree rings) are extremely limited in Australia, a situation common to most of the Southern Hemisphere [7]. The few existing records have been primarily in the tropical and sub-tropical north of the continent using tree rings and corals i.e., *Toona ciliata* ring widths in Queensland [8], coral luminescence from the Great Barrier Reef in Queensland [9,10], *Callitris intratropica* (now *C*. *columellaris*) ring widths in the Northern Territory combined with coral luminescence from the Great Barrier Reef [11,12]; with one reconstruction of past hydroclimate from ring widths of *C*. *columellaris* in the semi-arid southwest of the continent [13]. Our objective here is to fill a key knowledge gap by investigating the use of tree rings of the native cypress pine, *C*. *columellaris* growing in the semi-arid northwest of Australia to significantly extend the instrumental record back in time (i.e. > 100 years). In so doing, we aim to provide a longer-term context of hydroclimatic variability to help to understand both the significance and mechanisms behind the observed positive trend in summer precipitation in the region over recent decades.

## Methods

### Site description

**Fig 1:**
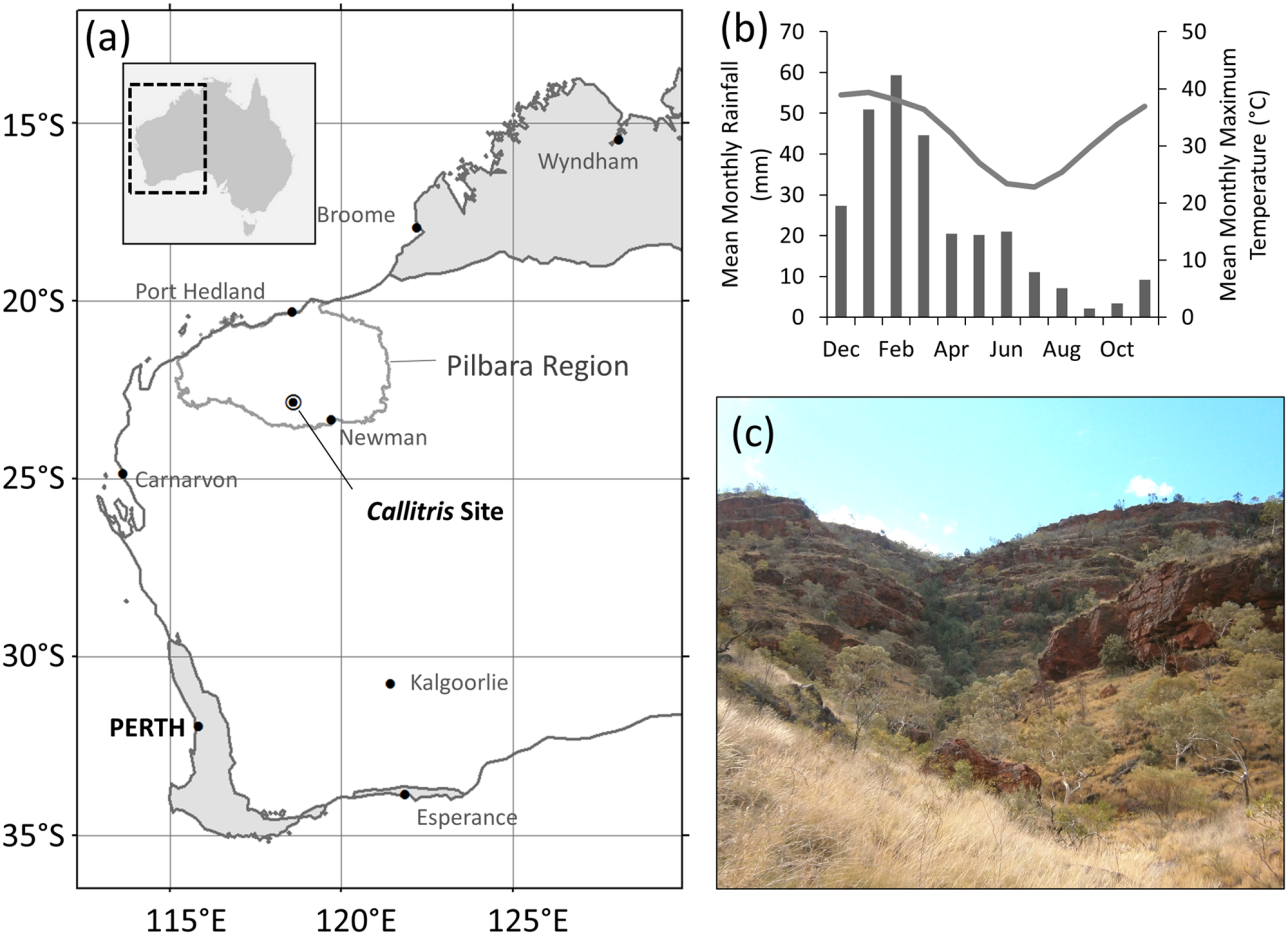
Location and climate of the *Callitris* tree-ring site. (a) Location of the *Callitris* site in the Pilbara region of northwest Australia; semi-arid and arid areas of Western Australia are shown in white; the temperate south-west and tropical north of Western Australia are shaded grey. (b) Long-term (1901–2012) mean monthly precipitation (bars) and maximum temperature (line) of the northwest region (i.e., CRU version 3.22 data area-averaged over 117–122°E, 21–26°S, see methods for details). (c) The tree ring site; a shallow, south-facing gully in the Hamersley Range. Dark green/grey trees visible in the centre of the image are *Callitris columellaris*, lighter-coloured trees are *Eucalyptus leucophloia*, and grasses in foreground and along the slopes are predominantly *Triodia* spp.

Our site is located in the eastern Pilbara region of northwest Australia (22.85°S, 118.62°E; Fig 1a), where a period of unusually high summer-autumn precipitation has been observed in recent decades. The Pilbara region is semi-arid with an average annual precipitation of 300–350 mm, which falls predominantly in the summer and autumn months (Dec–May, Fig 1b) but is highly variable on inter-annual timescales. Precipitation in the region is influenced by the Australian monsoon to the northeast and adjacent warm seas to the north, which generate tropical cyclones and tropical depressions [14]. During the dry season (Apr-Oct), winds in northwest Australia are dominated by the south-easterly Trade Winds, which bring rainfall to the tropical east coast of Australia but which tend to dry as they move over inland northern Australia, bringing stable, dry conditions to the northwest. During the monsoon wet season (Dec-Mar) the dominant winds shift to a north-westerly flow bringing moisture (heavy rain and thunderstorms) and squally winds to northern Australia [14]. Intense episodic precipitation events in summer and autumn are often associated with long-lived (>48 hours) closed lows (i.e., monsoonal depressions and tropical cyclones), which contribute more than half of all precipitation and >60% of extreme precipitation in inland semi-arid northwest Australia [4]. In contrast, short-lived (<12 hrs) lows generally contribute much less to precipitation [4]. Thunderstorms are common in the region during the summer and autumn months but their relative contribution to semi-arid north-west Australian precipitation remains poorly understood [14]. Cloud bands (extensive layers of cloud) that often stretch from the northwest to the southeast of the continent can bring sustained rainfall to northwest Australia, but generally during the dry season (Apr-Oct) [15]. The majority of precipitation in the Pilbara region falls as part of large-scale weather events (monsoonal depressions and tropical cyclones). Consequently, precipitation amount is strongly coherent over large spatial scales i.e., hundreds of kilometres (S1 Table).

### Sample collection

We sampled *Callitris columellaris* trees located in a south-facing, gently sloping, shallow gully in the Hamersley Range (Fig 1c). The gully is approximately 170 m wide at the widest point and all sampled trees were located within the drainage line or slopes of the gully (within an area of ca. 1.5 ha). The site is located on a pastoral lease. Permission to access the site was granted by the lease holders and samples were collected under a flora collection licence issued by the Western Australian Department of Parks and Wildlife. *Callitris columellaris* is not threatened or endangered; it is widespread across mainland Australia. *C*. *columellaris* has shallow roots and a conservative water use strategy, so its growth is highly responsive to precipitation [16]. Soils in the study area are skeletal and rocky and *C*. *columellaris* roots are often observed to follow the cracks of large boulders with little soil and no apparent access to groundwater. While precipitation is likely to be even across the small area of the study site, it is also likely that water availability to individual trees varies as a result of the terrain, which is reflected in differing growth rates among trees. However, this variability in water availability is generally consistent across time i.e., trees with greater access to water will consistently grow faster (wider rings) than trees with more restricted access to water. Consequently, while individual trees may vary in their magnitude of growth (ring width), the sampled population reveals similar patterns of inter-annual variation in relative ring width. *C*. *columellaris* is also fire sensitive, so populations in the Pilbara are generally confined to south-facing gullies which offer protection from the frequent fires of the surrounding floodplains and upper slopes.

We collected increment cores (5.15 mm diameter) from 35 live trees at heights of between 30 and 60 cm above ground level. Cores were not collected at the standard breast height (~1.3 m), because the trees often branch or become multi-stemmed above ~1 m height. *Callitris* trees are also generally slow growing in semi-arid climates and can take several decades to reach a height of 1.3 m [17]. Consequently, samples were collected at the lowest practicable height to capture as many years of growth as possible. A minimum of two cores were collected from each live tree preferably opposite (180°) to each other, or if this was not possible, at a minimum of 90° from each other for a total of 68 cores. We also collected stem sections from 24 standing or fallen dead trees that were mostly killed by fire in the summers of 2003/2004 or 2013/2014.

### Chronology development

Cores were prepared, crossdated and measured using standard dendrochronological techniques [18]. Crossdating was quality checked using the COFECHA program [19]. Half of the cores (34 of 68) were rejected because of excessive resin staining (15 cores), branch distortion (three cores), dry rot (four cores), indistinct rings (two cores), poor sample quality (e.g., cracked, missing sections, or off centre; three cores), or could not be crossdated due to suppressed growth with age (narrow rings; four cores). A further three cores were not included in the chronology because two other cores from the same tree were already included (a maximum of two cores per tree were included). Intra-annual and missing rings were encountered in several of the samples, but were generally easily identified during visual crossdating. We used 34 cores from 20 live trees and sections from seven dead trees to develop a ring-width chronology. Sections from other dead trees were rejected (17 of 24 trees) for a variety of reasons: they were long dead and could not be matched to the live trees (five trees); contained too few rings to contribute significantly to the chronology (three trees); could not be confidently crossdated due to slowed growth with age (many narrow and missing rings in the last century; seven trees); or were poorly correlated with the chronology (two trees).

The final selection of 41 series from 27 trees used to develop the ring-width chronology crossdated well, with high variability in ring width (μ = 0.72 mm, σ = 0.54 mm). We initially detrended our ring width series using the Friedman variable span smoother [20] but found that this method reduced the influence of wide rings in the most recent decades, which are associated with a period in the late 1990s and early 2000s when observed precipitation in the Pilbara region was particularly high. In order to retain this climatic signal in the ring-width chronology we used a signal free method [21] and a time-varying response (age-dependent) spline [22] to detrend our series using the RCSigFree program (http://www.ldeo.columbia.edu/tree-ring-laboratory/resources/software). Age-dependent splines are more flexible in the early part of the series and become progressively stiffer in the later part. Consequently, detrending ring-width chronologies using an age-dependent spline accounts for potential juvenile growth trends while retaining trends in the later years that are more likely to be related to trends in climate than to physiological or local site changes [22]. We used the ratio method to calculate our indices, which did not produce biased indices compared with the residual method [23] and provided a better fit to the climate data in terms of magnitude than the residual method.

Two widely-used parameters, the average correlation between series (RBAR) and expressed population signal (EPS), were used to assess the quality of our chronology. RBAR provides an indication of chronology signal strength (common variance) and is independent of sample size [24]. The EPS provides an indication of the likely loss of reconstruction accuracy as a function of RBAR and sample size, measuring how well the finite-sample chronology compares with the theoretical population chronology based on an infinite number of trees [25]. While there is no formal level of significance for EPS, the value of 0.85 is generally accepted as a reasonable limit for the chronology to remain reliable. Running RBAR and EPS statistics were calculated for 51 year intervals of the chronology with 25 year overlaps to assess the stability of signal strength as chronology replication diminished back in time. Only that portion of the chronology where the EPS exceeded 0.85 was used for climate reconstruction.

**Fig 2:**
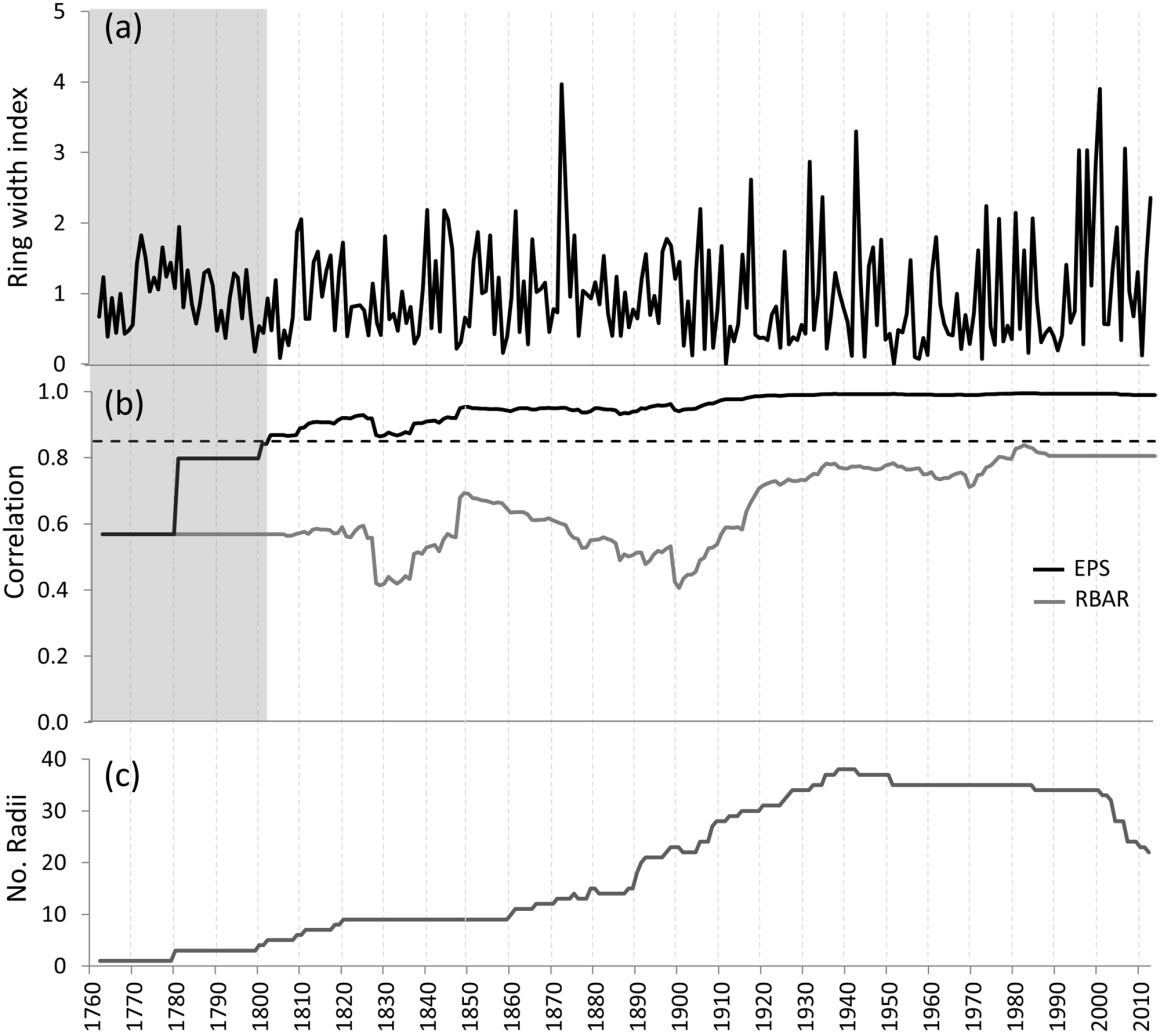
The *Callitris* ring-width chronology from the Pilbara region in northwest Australia. (a) Ring-width indices, (b) measures of signal strength, the $\overset{-}{R}$ (RBAR) and the expressed population signal (EPS), and (c) sample depth. The black dashed line indicates the accepted level of confidence of the EPS (0.85). The shaded area indicates the point (1802) when the EPS drops below 0.85 and therefore when the chronology is considered to be less reliable.

The final *C*. *columellaris* ring-width chronology was ca. 250 years long (CE 1762 to 2012) (Fig 2a). However, changes in running EPS suggest that the chronology, and therefore a reconstruction based on this chronology, is reliable only after CE 1802 (EPS > 0.85, Fig 2b). Consequently, we only consider here the outer 210-year period, CE 1802–2012, of the reconstruction in our findings.

### Climate data

We obtained the Climatic Research Unit’s (CRU) precipitation and maximum temperature data (version 3.22) and the self-calibrating Palmer drought severity index (scPDSI, version 3.22; [26]) data from KNMI Climate Explorer (http://climexp.knmi.nl/). Precipitation data from weather stations in the Pilbara region are strongly correlated (r > 0.80) with regionally-averaged CRU precipitation data (S1 Table) showing that the CRU gridded data provide a reliable representation of instrumental climate records in the region. The scPDSI is a widely used measure of drought or more specifically, soil moisture availability [27] and is based on a water balance model calculated using instrumental records of precipitation and temperature and a general soil water holding capacity parameter. Data prior to 1910 were excluded from the calibration and reconstruction because instrumental precipitation records were limited across the Pilbara region during this period, making the gridded data relatively unreliable.

We also obtained data for broad-scale climate indices known to influence northwest Australian climate from KNMI Climate Explorer (http://climexp.knmi.nl/). The Southern Annular Mode (SAM) is a measure of the difference in normalised monthly mean sea level pressure (MSLP) between 40°S and 65°S and is a measure of north-south hemispheric-wide migrations in westerly winds [28]. During a positive phase, the belt of westerly winds moves southward, generally resulting in weaker than normal westerly winds, higher pressure, fewer storm systems and less winter precipitation over southwest and southeast Australia [29], but has a spatially and seasonally variable influence on precipitation across the rest of the continent [30]. We obtained the British Antarctic Survey’s SAM index data (1957–2014) from Climate Explorer.

The El Niño-Southern Oscillation (ENSO) is often measured by the Southern Oscillation Index (SOI). The SOI is calculated from fluctuations in the air pressure difference between Tahiti and Darwin, Australia. Strong negative (positive) values of SOI for several months or more typically indicate El Niño (La Niña) phases of the ENSO. We used the Climatic Research Unit’s SOI data (1866–2014) available from Climate Explorer. We also used the Niño 3.4 and Niño 4 indices of the ENSO, which are based on area-averaged sea surface temperatures in the Niño 3.4 (5°N–5°S, 170°W and 120°W) and Niño 4 regions (5°N–5°S, 160°E–150°W). Niño 3.4 and 4 data [31] for the period 1856–2013 were obtained from Climate Explorer.

The Ningaloo Niño (Niña) is a recently identified phenomenon of anomalously warm (cool) sea surface temperature off the western Australian coast [32–34], which has been linked to increased (decreased) rainfall in the northwest of Australia [35]. The Ningaloo Niño index is calculated as area-averaged SST anomalies. The area used to calculate the Ningaloo Niño index varies, but is generally between 108° and 116°E and between 22° and 32°S [32–35]. We obtained sea surface temperature data from the NOAA extended reconstructed sea surface temperature dataset (ERSST, version 3b; 1854–2014), available from Climate Explorer. We calculated a Ningaloo Niño index from anomalies of sea surface temperatures averaged over the region: 108°–116°E and 22°–28°S.

The Indian Ocean Dipole (IOD) is an ENSO-like coupled ocean-atmosphere phenomenon in the equatorial Indian Ocean. The IOD typically occurs between May and November but peaks between June and October [30]. The IOD is often measured by the Dipole Mode Index (DMI), which is calculated as the difference in the area-averaged anomalies of sea surface temperature between the tropical west Indian Ocean (10°N–10°S, 50°E–70°E) and the tropical southeast Indian Ocean (0°–10°S, 90°E–110°E). We used the DMI based on HadISST1 (1870–2014) available from Climate Explorer.

### Tree growth–climate relationships

**Fig 3:**
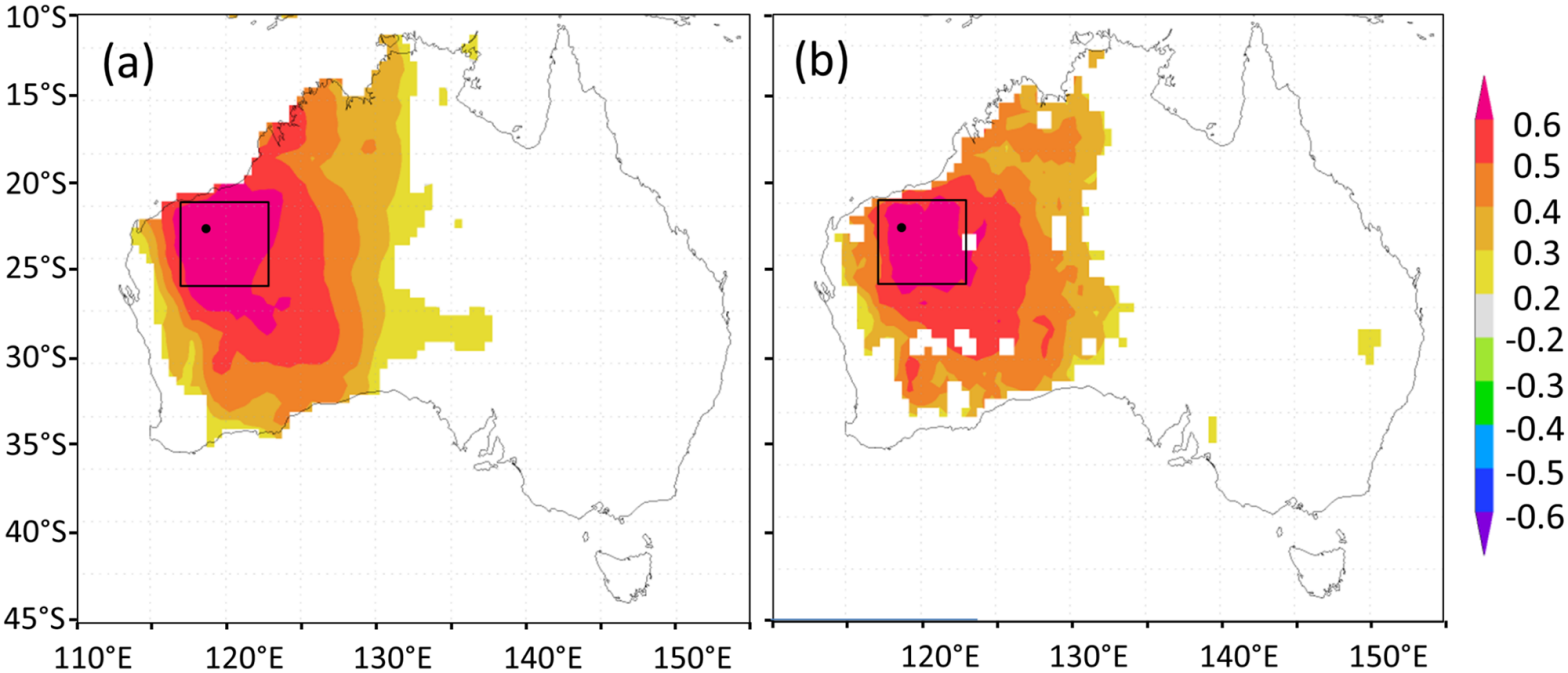
Significant (p < 0.05) correlations between the *Callitris* ring-width chronology and (a) summer-autumn (Dec–May total) precipitation and (b) autumn (Mar–May averaged) scPDSI across Australia (1910–2011). Correlation maps were produced in Climate Explorer (climexp.knmi.nl). Precipitation and scPDSI data are CRU version 3.22 0.5° gridded datasets area-averaged over the 5° study region (117–122°E, 21–26°S).

We used simple correlation analyses to identify climate signals in the detrended ring-width chronology using the PCReg program (http://www.ldeo.columbia.edu/tree-ring-laboratory/resources/software). Correlations between the ring-width chronology and all gridded climate variables were strong and spatially coherent over broad areas (up to 5x5°) (Fig 3). Consequently, we averaged the gridded data (in Climate Explorer) over a 5 x 5° (117°E–122°E, 21°S–26°S; Fig 3) area to obtain a dataset of regional climate. We tested for significant correlations between the ring-width chronology and the regionally-averaged maximum temperature, precipitation amount and scPDSI over an 18-month dendroclimatic window [24] including the current and previous growth seasons.

The Pilbara ring-width chronology is strongly correlated with regional precipitation and scPDSI in all months of the summer-autumn season (Table 1) and is also strongly correlated with mean summer-autumn regional maximum temperature (Dec–May, r = -0.65; 1910–2011; *p* <0.0001). The summer-autumn (Dec-May) mean of monthly precipitation is highly correlated with the summer-autumn mean of monthly maximum temperatures (r = -0.82; 1910–2011; *p* <0.0001) and autumn (Mar-May) mean of monthly scPDSI (r = 0.91; 1910–2011; *p* <0.0001). We chose total summer-autumn precipitation as the target climate variable and season to reconstruct since this variable had the highest correlation with the ring-width chronology (Table 1). This is also the same combined season that has shown an unusual and significant increasing trend in precipitation in northwest Australia over the last several decades. We also tested for correlations between the ring-width chronology and regional climate and indices of broad-scale circulation patterns (SAM, SOI, Niño 4, and DMI).

**Table 1:** Significant correlations (α < 0.05) between the *Callitris columellaris* ring-width chronology and monthly and seasonal total precipitation and average scPDSI (1910–2012) during the growing season of *Callitris columellaris* (ca. Dec–May) in semi-arid northwest Australia.

		Total Rainfall	Average PDSI
**Monthly**	December	0.45	0.41
	January	0.54	0.58
	February	0.41	0.62
	March	0.55	0.72
	April	0.39	0.72
	May	0.16	0.66
**Seasonal**	Summer (Dec-Feb)	0.67	0.57
	Autumn (Mar-May)	0.64	0.73
**Growing Season**	Dec-May	0.81	0.68

Precipitation and scPDSI are the CRU version 3.22 0.5° gridded datasets area-averaged over the 5° study region (117–122°E, 21–26°S, Fig 1). All *p*-values associated with correlation (r) values are <0.0001 except where NS is shown. NS indicates non-significant correlation values (i.e., *p* > 0.05).

### Reconstruction model

A linear model was used to reconstruct summer-autumn precipitation as a function of the detrended ring-width chronology. Split-period calibration/verification analysis [24,36] was used to test the robustness of the model. Two rigorous tests of fit, the reduction of error (RE) and the coefficient of efficiency (CE) were used to verify the model. Our model passed both the CE and RE tests (strongly positive [24]) indicating that the model was skilful in reconstructing observed variations (Table 2). We then used the full period (1910–2012) of observed data to develop the final linear model to reconstruct summer-autumn precipitation back to 1802. There was no significant autocorrelation in the residuals of the final linear model (p > 0.05, Durbin Watson test, dwtest in the R package; lmtest, [37]). The final model accounts for 66% of the variance in the observed summer-autumn precipitation (Table 2).

**Table 2:** Calibration and verification statistics for the reconstruction of summer-autumn (Dec–May) total precipitation in semi-arid northwest Australia from the *Callitris columellaris* ring-width chronology.

Calibration Period	r	R^2^_Adj._	Verification Period	r	RE	CE
Late (1960–2012)	0.85	0.71	1910–1959	0.75	0.60	0.48
Early (1910–1959)	0.75	0.55	1960–2012	0.85	0.70	0.65
Full (1910–2012)	0.81	0.66	-	-	-	-

Precipitation is the CRU 3.22 0.5° gridded dataset area-averaged over the 5° study region (117–122°E, 21–26°S, Fig 1). r is the Pearson correlation coefficient, R^2^_Adj_ is the coefficient of determination adjusted for the number of terms in the model, RE is the Reduction of Error, CE is the Coefficient of Efficiency. All *p*-values associated with correlation values (r) are < 0.0001.

We rescaled the variance of the reconstructed data to match the variance of the instrumental data, which allows comparisons to be made between extreme events in the past and those in the instrumental or observed record. We used 20-year loess smoothing to highlight ca. decadal-scale trends and calculated 95% prediction intervals for the linear models in R 3.1.0 [38].

## Results and Discussion

### Reconstruction of hydroclimate

**Fig 4:**
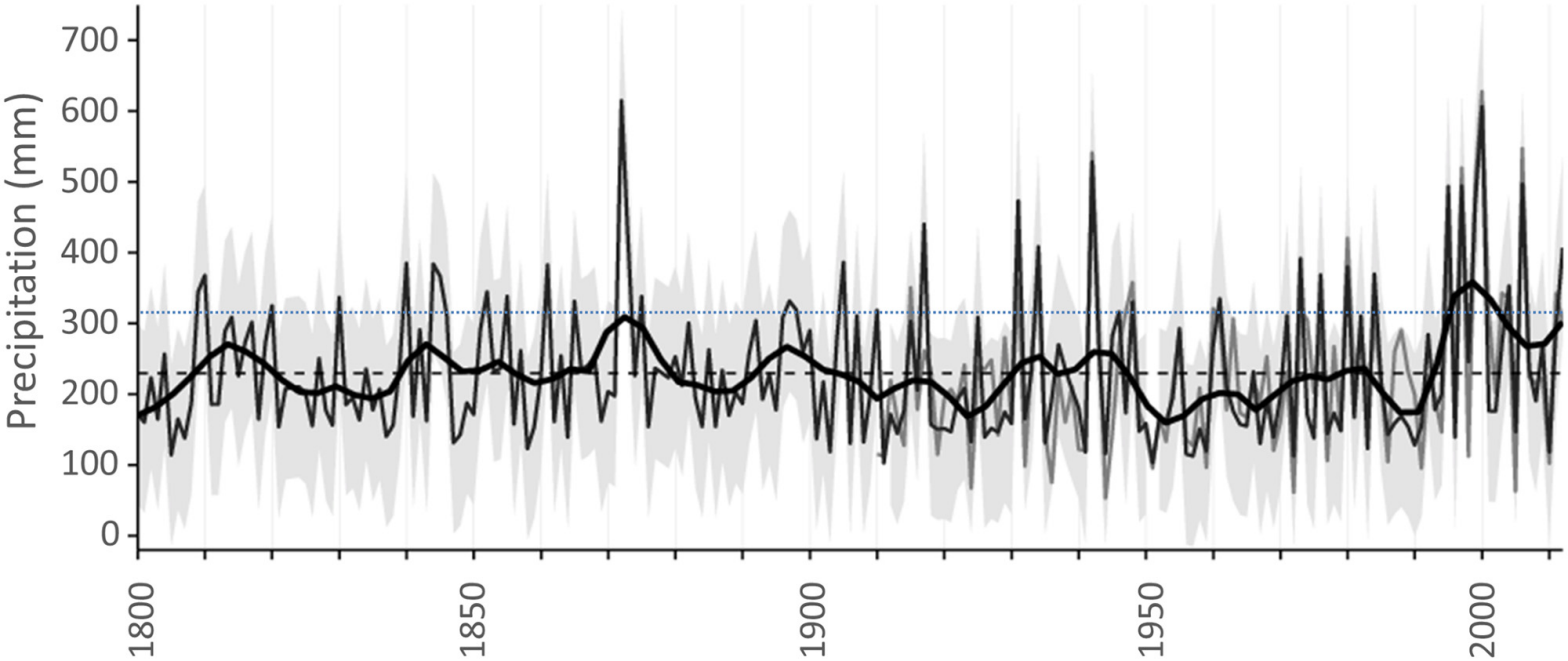
Temporal variability of summer-autumn precipitation in semi-arid northwest Australia over the last two centuries. Observed precipitation is shown by the mid-grey line and reconstructed precipitation is shown by the dark grey line. A 20-year loess smoothing curve is shown by the solid black line; the long-term (1802–2012) mean is shown by the dashed black line; 95% prediction intervals (fitted with the predict function in R 3.1.0) are indicated by light grey shading. The dotted blue line indicates where scPDSI is equal to zero and highlights the recent period (1995–2002) when scPDSI was greater than zero.

Ring widths of *Callitris columellaris* in northwest Australia are highly responsive to precipitation, which is consistent with knowledge of the opportunistic growth of the species in other climates in Australia [13,16,39]. The maximum reconstructed precipitation amount exceeded 600 mm and reconstructed precipitation in the wettest years matched the observed data well in terms of magnitude (Fig 4a). This finding suggests that tree growth does not reach an asymptote (i.e., become limited by factors other than water availability) in high precipitation years, at least within the observed range of precipitation. However, our reconstruction tends to underestimate the magnitude of severe summer-autumn drought (ca. < 100mm of precipitation). *Callitris columellaris* trees are highly drought resistant; their growth is constrained only when soil moisture content becomes extremely low (transpiration ceases when soil moisture approaches air dryness; <4%) and they can persist in an state of arrested growth for prolonged periods of drought [39]. In our study, in years when observed summer-autumn precipitation was less than ca. 100 mm many trees did not produce a discernible growth ring, resulting in extremely small mean ring-width measurements in those years (< 0.1 mm). Consequently, the minimum reconstructed summer-autumn precipitation (ca. 100 mm in 1911) overestimates the minimum observed precipitation (53 mm in 1944). Hence, the ring-width chronology is likely a more accurate record of the magnitude of extreme wet periods rather than of extremely dry periods i.e., <100 mm total summer-autumn precipitation.

### Temporal patterns of reconstructed precipitation

The reconstruction of precipitation reveals inter-annual to multi-decadal-scale variation in hydroclimate of semi-arid northwest Australia over the last 210 years (Fig 4). In particular, the reconstruction exhibits fluctuations from prolonged periods (10–30 years) of below average precipitation to shorter periods (typically 5–10 years) of above average precipitation (Fig 4). Years with extreme summer-autumn precipitation (> ca. 400 mm) have occurred irregularly over the last two centuries (Fig 4) and are important drivers of the boom (and bust) behaviour of productivity in semi-arid and arid Australia [40–42]. Large and intense precipitation events are also particularly important for recharging the intermittent streams and floodplains of the semi-arid northwest [43]. Indeed, the timing of extreme precipitation events during the 20^th^ Century determined from our tree-ring based reconstruction (e.g., 1942, 1995, 1997, 1999, 2000, 2006) corresponds with the largest and most prolonged floods on the Fortescue Marsh, the largest wetland in inland northwest Australia [44].

Our reconstruction shows that the most recent two decades (1995–2012) were unusually wet compared to the previous two centuries (Fig 4; 20-year loess curve exceeded the long-term (1802–2012) standard deviation of 321 mm). This finding is consistent with a recent detailed reconstruction of the expression of precipitation on the Fortescue Marsh in the Pilbara region [44]. This anomalous wet period is attributed to an unusually high frequency of years with extreme summer-autumn precipitation (> ca. 400 mm). Five of the 10 wettest (> ca. 400 mm) years in the last 210 years occurred in the last two decades (i.e., 1995, 1997, 1999, 2000 and 2006), all of which are associated with one or more tropical cyclones crossing the northwest Australian coast between December and March.

The unusually high observed summer-autumn precipitation in semi-arid northwest Australia over the last two decades (mean of 311 mm for 1995–2012 compared with a mean of 205 mm for 1910–1994) has been mostly attributed to both a high frequency of tropical cyclones (peaking at 2.7 cyclones/year in 1991–2001 [45]) as well as an increase in the intensity of precipitation (i.e., > 0.2mm rain/low day/year, 1989–2009 [4]) from tropical cyclones and other closed low pressure systems [4,46]. Reported linear trends in tropical cyclone frequency in northwest Australia vary depending on the period examined. While there appears to have been a decline in tropical cyclone frequency between the beginning of instrumental tropical cyclone records in 1970 and the late 1990s [47], there is no clear evidence of a change in the frequency of tropical cyclones over the full record period (e.g., 1970–2008; [48]). However, there is general consensus that the frequency of the most severe tropical cyclones (minimum pressure of 970 hPa or lower) has increased in northwest Australia over the last 40 years [47,49,50].

Importantly, our finding is in contrast to a recent proxy record, based on the δ^18^O of a single speleothem on the semi-arid northwest Australian coast, which while not correlated with precipitation, suggests that tropical cyclone activity (as measured by a cyclone activity index, CAI) in western Australia has been lower in the last five decades (since 1960) than at any point in the last ca. 1500 years [51]. While the CAI may have declined along the semi-arid northwest coast where the site was located; our results and others (i.e., [52]) suggest this finding should not be extrapolated to the rest of northwest Australia and is particularly not applicable to inland northwest Australia. The discrepancy between our findings from inland and those from coastal semi-arid northwest Australia [51] (ca. 500 km apart) highlights the need for greater spatial resolution and coverage of climate proxies in Australia. Our findings, along with previous chronologies and reconstructions developed from *Callitris columellaris* elsewhere in Australia [11–13] have shown this species is an excellent proxy for reconstructing past hydroclimatic variability. *Callitris columellaris* (and other suitable species in the genus) are also widespread across Australia and therefore have significant potential for extending climate records and improving the spatial resolution of records of past climates across the continent.

### Drivers of northwest Australian precipitation

Given that the strongest regional climate signal in the chronology is summer-autumn precipitation, we expected that broad-scale circulation patterns that drive summer-autumn precipitation in the semi-arid northwest would also be correlated with the ring-width chronology. While ENSO has a strong influence on precipitation patterns in eastern Australia, its influence on precipitation in western Australia has been much more variable [30]. Consequently, we found ENSO was only weakly to moderately correlated with our ring width chronology and summer-autumn regional precipitation and scPDSI (Table 3).

**Table 3:** Correlations between broad-scale climate drivers and the *Callitris columellaris* ring-width chronology and regional precipitation and scPDSI in northwest Australia.

			RW Chronology		Precipitation		PDSI		
	Index	Season	r	p-value	r	p-value	r	p-value	years (n)
**SAM**	SAM	Dec-May	0.50	0.0001	0.40	0.0021	0.47	0.0003	1957–2011
**ENSO**	Niño4	Dec-May	-0.22	0.0302	-0.21	0.0296	-0.31	0.0016	1910–2011
	SOI	Dec-May	0.20	0.0460	0.23	0.0221	0.29	0.0043	1910–2011
	Niño3.4	Dec-May	-0.20	0.0435	-0.21	0.0378	-0.29	0.0036	1910–2011
**IOD**	DMI	Dec-May	0.17	NS	0.04	NS	-0.01	NS	1910–2011
	DMI	Jun-Oct	0.11	NS	-0.33	0.0005	-0.18	NS	1910–2012
**NN**	NNI	Dec-Feb	0.45	<0.0001	0.41	<0.0001	0.55	<0.0001	1910–2011

Regional precipitation and scPDSI are the CRU 3.22 0.5° gridded datasets area-averaged over the 5° study region (117–122°E, 21–26°S, Fig 1). SAM is the Southern Annular Mode, ENSO is the El Niño Southern Oscillation, IOD is the Indian Ocean Dipole, NN is the Ningaloo Niño, SOI is the Southern Oscillation Index, DMI is the Dipole Mode Index, and NNI is the Ningaloo Niño index. NS indicates non-significant correlation values (i.e., *p* > 0.05).

**Fig 5:**
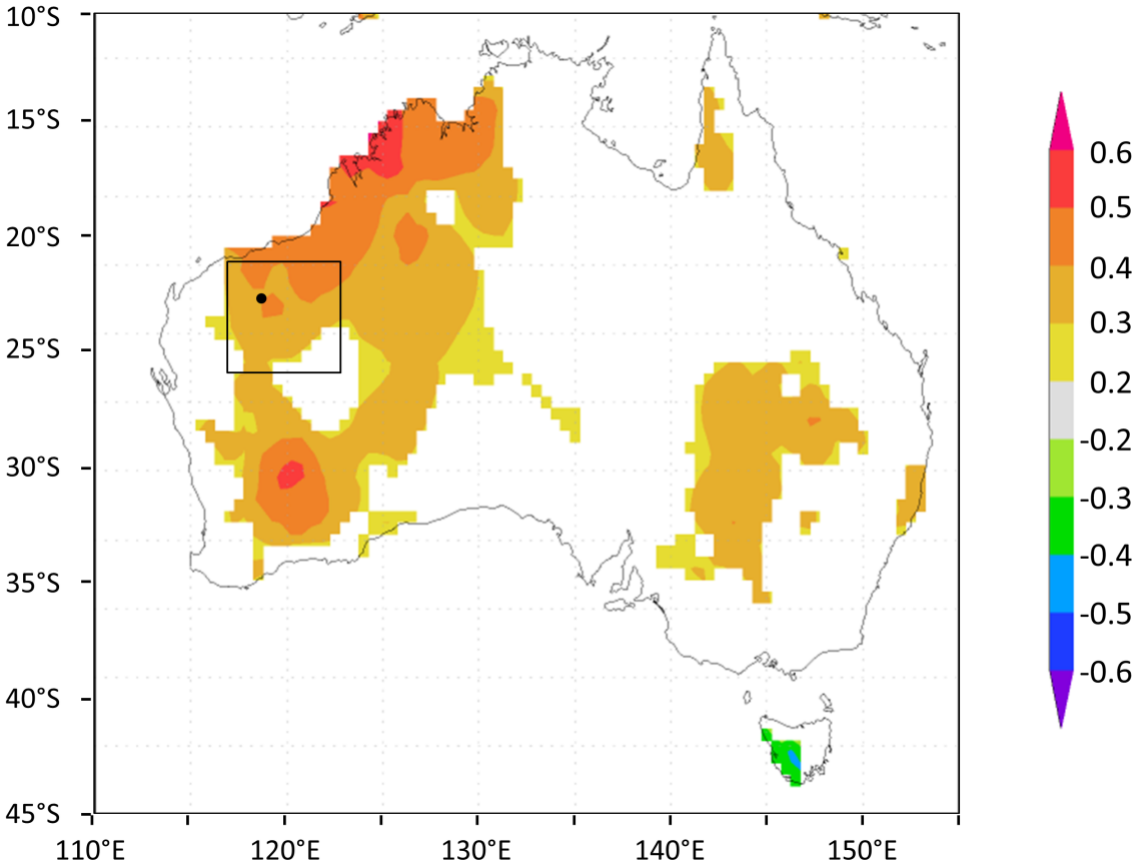
Significant (p < 0.05) correlations between the summer-autumn (Dec–May averaged) Southern Annular Mode (SAM) and precipitation across Australia (1957–2012). Image produced in Climate Explorer (climexp.knmi.nl). SAM data are from the British Antarctic Survey and precipitation data are CRU version 3.22 0.5° gridded data area-averaged over the 5° study region (117–122°E, 21–26°S).

IOD events generally occur in winter-spring (Jun–Oct) and are known to strongly influence northwest Australia precipitation patterns [30]. While the IOD has a strong relationship with winter-spring precipitation in the eastern Pilbara (Table 3), it is not related to summer-autumn precipitation and consequently does not appear to be a strong determinant of tree growth in the region. However, sea surface temperatures near the western Australian coast (“Ningaloo Niño” region) are strongly and positively correlated with our ring-width chronology and summer-autumn regional precipitation and scPDSI (Table 3), suggesting a link between Indian Ocean climate and northwest Australian hydroclimate in this season. Ningaloo Niño events are promoted by wind-evaporation-SST feedbacks: cyclonic anomalies act to reduce the surface wind speed and increase SSTs thereby driving increased rainfall in northwest Australia and stronger cyclonic anomalies [32,35]. The Ningaloo Niño also interacts with ENSO, such that La Niña conditions in the Pacific Ocean can enhance the strength of the Ningaloo Niño [32],

Interestingly, of the climate indices tested, the SAM was most strongly correlated with our ring-width chronology (r = 0.49), as well as with regional precipitation and scPDSI in summer and autumn (Table 3). The SAM is usually considered for its influence on southern Australian precipitation, where a positive phase of the SAM in winter usually results in drier conditions in southwest and southeast Australia [29]. However, a positive phase of the SAM in summer and autumn has also recently been linked to wetter conditions in the subtropics (approximately 20–35°S [53]), particularly in northwest Australia (Fig 5). A positive SAM during summer and autumn drives eddy-induced divergent meridional circulation in the subtropics and a poleward shift of the subtropical dry zone resulting in higher precipitation in the subtropics including inland semi-arid northwest Australia [53].

### Drivers of the recent wet period in northwest Australia

Several mechanisms have been suggested to explain the recent period of high summer-autumn precipitation and high tropical cyclone activity in northwest Australia. Increased concentrations of aerosols, particularly from the Asian region have been suggested as a potential driver of the increased precipitation [54,55] and tropical cyclone frequency [56] in northwest Australia based on simulation models. However, the actual impact of aerosols on Australian precipitation remains unclear [57].

**Fig 6:**
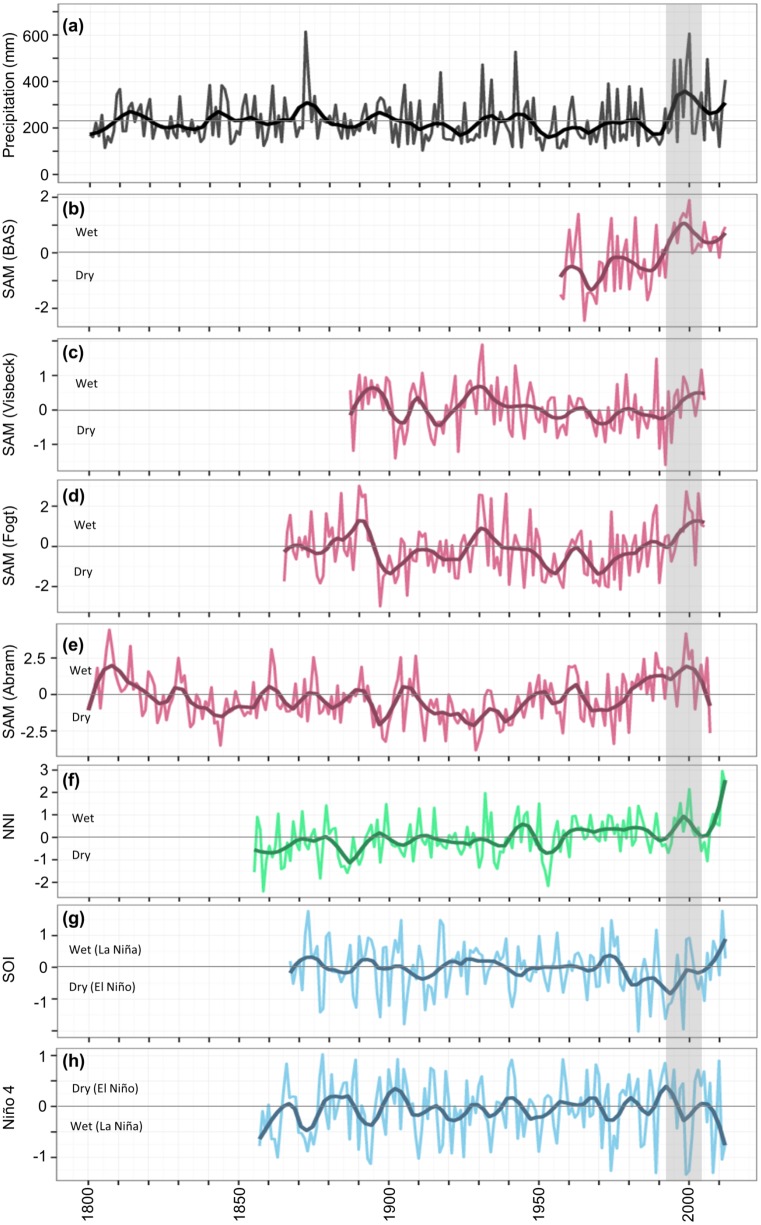
Variation in reconstructed northwest Australian precipitation, the Southern Annular Mode, The Ningaloo Niño and the El Niño Southern Oscillation over the last two centuries. (a) Reconstructed precipitation in semi-arid northwest Australia; the Southern Annular Mode (SAM) (b) as an observation-based index calculated by the British Antarctic Survey (BAS) and two instrumental reconstructions by (c) Visbeck [72] and (d) Fogt [70,71] and (e) a tree-ring based reconstruction of the SAM [65]; (f) the Ningaloo Niño index (NNI, see text for definition); and the El Niño Southern Oscillation (ENSO), as (g) the Southern Oscillation Index (SOI) and (h) the Niño 4 SST index. Darker lines in each plot are 20-year loess smoothing curves. “Wet” and “Dry” labels indicate the typical effect of the SAM, NN and ENSO on summer-autumn precipitation in inland northwest Australia. The shaded box highlights the timing of the recent wet period in inland semi-arid northwest Australia.

ENSO is a known driver of tropical cyclone activity (and subsequent precipitation) in the western Australian region, where tropical cyclone activity is enhanced (suppressed) in La Niña (El Niño) years [58]. However, the relationship between ENSO (as SOI) and tropical cyclone activity is limited to moderate-severity cyclones (between 970 and 990 hPa), which have not significantly increased in frequency. The observed increase in the frequency of tropical cyclones is restricted to the most severe category (minimum 970 hPa or lower), which is not attributable to ENSO [47]. Long-term (50-year) trends in ENSO are also unable to explain the positive trend in summer-autumn precipitation (from both tropical cyclone and non-tropical cyclones sources) in northwest Australia [57]. However, over the last two decades (since ca. 1990), there has been an increasing trend in the SOI (CRU, climex.knmi.nl) and dominance of La Niña conditions [59], which coincides with the period of high precipitation in northwest Australia in the last two decades (Fig 6).

Since the late 1990s there has been a marked increase in the occurrence of Ningaloo Niño events [33,60], coincident with the period of increased summer-autumn precipitation in northwest Australia. This trend in the Ningaloo Niño has been linked to the Interdecadal Pacific Oscillation, which has shifted to its negative phase over the same period [33]. A reconstruction of SSTs from corals shows that SSTs off the mid-west Australian coast have been increasing over the last two centuries and that recent SSTs have been higher than in at least the last 215 years [61,62]. It has been suggested that the anomalously warm conditions in the Ningaloo Niño region since the 1990s may have contributed to the anomalous precipitation in northwest Australia through its influence on convective rainfall and cyclonic anomalies, although the mechanistic links between regional SSTs off the west coast and precipitation over land need to be clarified further [60].

Since the early 1990s there has also been a shift in the Southern Hemisphere atmospheric circulation and an increase in the dominance of the positive phase of SAM [63] (Fig 6). The high frequency and dominance of the positive phase of SAM in austral summer over the last few decades is unprecedented in the last 600–1000 years [64,65] and is consistent with forcing by the Antarctic Ozone Hole [29,63,66]. The dominance of the positive phase of the SAM has contributed to half of the winter precipitation reduction observed in southwest Australia over recent decades [67] and has also been associated with increased summer precipitation in the latter half of last century in inland central Western Australia [59,68]. The SAM has also been linked to increasingly arid conditions in the South American Andes [69]. The positive trend in the SAM coincides with and is also likely a major driver of the period of high summer-autumn precipitation observed over recent decades in semi-arid northwest Australia [53] (Fig 6).

Our findings suggest that the SAM has played a role in driving summer-autumn hydroclimatic variability in semi-arid northwest Australia over at least the last century. While the correlation between our ring width chronology and the SAM reported here (Table 3) is based only on the 1957–2012 observational record of SAM, our ring width chronology is significantly correlated with instrument-based reconstructions of the autumn SAM by Fogt [70,71] (r = 0.32, *p* = 0.0007, 1900–2005; available from: http://polarmet.osu.edu/ACD/sam/sam_recon.html) and Visbeck [72] (r = 0.24, *p* = 0.0151, 1900–2005) and a tree-ring based reconstruction of the SAM by Abram and others [65] (r = 0.26, *p* = 0.0068, 1900–2007) over the 20^th^ Century (Fig 6). Tree-ring based reconstructions provide insight into the behaviour of the SAM over the last 600–1000 years [64,65]; however, it is likely that the relationship between the SAM and northwest Australian hydroclimate was not stable prior to the most recent century (i.e., there was no significant correlation between reconstructed precipitation in northwest Australia and the SAM reconstruction by Abram prior to 1900) so we cannot make inferences about hydroclimatic variability in northwest Australia over longer timescales from these reconstructions. However, the influence of SAM on northwest Australian precipitation is expected to continue into the near future, so projected changes in the behaviour of the SAM are likely to have significant implications for precipitation patterns in northwest Australia. While the ozone hole is expected to recover over the next few decades, the positive trend in the SAM is projected to continue in the austral summer and also to increase in other seasons as a result of continued increasing concentrations of greenhouse gases [53,63]. Our findings suggest that if there are further increases in the frequency and dominance of the positive phase of the SAM, this may be coupled with further increases in summer-autumn precipitation in semi-arid northwest Australia.

## Conclusions

The reconstruction of Dec–May total precipitation (i.e., for the austral summer-autumn) from *Callitris columellaris* tree rings represents a significant extension of hydroclimatic data back in time for the semi-arid northwest of Australia and an important contribution to records of past climates in the Southern Hemisphere over the last two centuries. Our findings highlight the enormous potential of tree rings of *Callitris columellaris* (and likely other long-lived species in the genus) to provide insights into past climate variability throughout mainland Australia.

Our reconstruction shows that the most recent two decades have been unusually wet in semi-arid northwest Australia compared with the past two centuries. It further suggests that this period of unusually high summer-autumn precipitation has likely been driven by an increase in the dominance and frequency of the positive phase of the SAM, highlighting the potential impact of anthropogenic-driven changes in the behaviour of the SAM on the hydroclimate of northwest Australia.

## Supporting Information

S1 Table Correlations and distances among individual precipitation stations and gridded precipitation data in the Pilbara region, northwest Australia. Note: Precipitation data are the CRU 3.22 0.5° gridded data area-averaged over the 5° study region (117–122°E, 21–26°S). Correlations are Pearson correlation coefficients. All *p*-values associated with correlation values are < 0.0001.
(DOCX)
