# Supplementary material for: Tree Rings Show Recent High Summer-Autumn Precipitation in Northwest Australia Is Unprecedented within the Last Two Centuries
Source: PLoS One. 2015 Jun 3;10(6):e0128533. doi: 10.1371/journal.pone.0128533 (PMC4454581; doi:10.1371/journal.pone.0128533)
Supplement: S1 Table — Note: Precipitation data are the CRU 3.22 0.5° gridded data area-averaged over the 5° study region (117–122°E, 21–26°S). Correlations are Pearson correlation coefficients. All p-values associated with correlation values are < 0.0001. (DOCX) [file pone.0128533.s001.docx]

|  |  | **Distances (km)** | | | | | | | | | | | | |  |
| --- | --- | --- | --- | --- | --- | --- | --- | --- | --- | --- | --- | --- | --- | --- | --- |
|  |  | CRU Precip | Mt Florance | Hamersley | Mulga Downs | Wittenoom | Marandoo | Packsaddle | Marillana | Rhodes Ridge | Bonney Downs | Turee Creek | Prairie Downs | Marymia | Year Range |
| **Correlations** | Mt Florance | 0.83 | - | 57 | 71 | 70 | 96 | 150 | 185 | 212 | 219 | 218 | 251 | 421 | 1886-2013 |
|  | Hamersley | 0.84 | 0.88 | - | 83 | 68 | 58 | 125 | 182 | 195 | 233 | 179 | 226 | 387 | 1910-2013 |
|  | Mulga Downs | 0.85 | 0.87 | 0.87 | - | 20 | 69 | 92 | 113 | 144 | 151 | 169 | 187 | 361 | 1897-2013 |
|  | Wittenoom | 0.89 | 0.88 | 0.88 | 0.94 | - | 50 | 81 | 118 | 142 | 165 | 156 | 181 | 353 | 1950-2013 |
|  | Marandoo | 0.80 | 0.83 | 0.91 | 0.87 | 0.90 | - | 68 | 133 | 139 | 194 | 123 | 167 | 329 | 1965-1998 |
|  | Packsaddle | 0.90 | 0.85 | 0.86 | 0.91 | 0.89 | 0.87 | - | 78 | 72 | 150 | 80 | 102 | 272 | 1989-2002 |
|  | Marillana | 0.83 | 0.70 | 0.69 | 0.77 | 0.77 | 0.71 | 0.89 | - | 52 | 74 | 133 | 102 | 273 | 1936-2013 |
|  | Rhodes Ridge | 0.90 | 0.81 | 0.79 | 0.86 | 0.86 | 0.82 | 0.92 | 0.89 | - | 118 | 93 | 50 | 225 | 1971-2013 |
|  | Bonney Downs | 0.81 | 0.66 | 0.71 | 0.73 | 0.71 | 0.70 | 0.79 | 0.76 | 0.80 | - | 206 | 161 | 317 | 1907-2013 |
|  | Turee Creek | 0.83 | 0.70 | 0.74 | 0.74 | 0.77 | 0.73 | 0.78 | 0.71 | 0.77 | 0.67 | - | 77 | 208 | 1920-2013 |
|  | Prairie Downs | 0.87 | 0.79 | 0.78 | 0.79 | 0.81 | 0.74 | 0.84 | 0.77 | 0.85 | 0.71 | 0.89 | - | 176 | 1968-2013 |
|  | Marymia | 0.81 | 0.63 | 0.63 | 0.63 | 0.69 | 0.59 | 0.78 | 0.64 | 0.76 | 0.62 | 0.72 | 0.74 | - | 1972-2013 |
